# Endocrine disorders after heart transplantation: national cohort study

**DOI:** 10.1186/s12902-020-0533-6

**Published:** 2020-04-20

**Authors:** Matej Rakusa, Bojan Vrtovec, Gregor Poglajen, Andrej Janez, Mojca Jensterle

**Affiliations:** 1 Department of Endocrinology, Diabetes and Metabolic Disease, University Medical Centre Ljubljana, Ljubljana, Slovenia; 2 Department of Internal Medicine, Faculty of Medicine, University of Ljubljana, Ljubljana, Slovenia; 3 Advanced Heart Failure and Transplantation Programme, Department of Cardiology, University Medical Centre Ljubljana, Ljubljana, Slovenia

**Keywords:** Heart transplantation, Endocrine disorders, Osteoporotic fractures, Multiple endocrine disorder

## Abstract

**Background:**

Endocrine disorders in patients after heart transplantation (HT) remain understudied. We aimed to assess endocrine profiles and management of HT recipients in the early post- transplant period.

**Methods:**

We conducted a retrospective cohort study on 123 consecutive HT recipients in the Advanced Heart Failure and Transplantation Programme between 2009 and 2018. All recipients had per-protocol endocrine follow-up within the first postoperative year. The median time to first post-transplant endocrine follow-up was 3 months (IQR 2–4). We assessed the incidence of vitamin D deficiency, bone mineral density, history of low energy fractures, hypogonadism in male recipients, posttransplant diabetes mellitus, and thyroid and parathyroid function.

**Results:**

We enrolled 22 women and 101 men of median age 57 years (IQR 50–63). Post-transplant diabetes mellitus developed in 14 patients (11.4%). 18 of 25 patients (14.6%) with preexisting type 2 diabetes mellitus required intensification of antidiabetic therapy. 38 male patients (40.4%) had hypogonadism. 5 patients (4.6%) were hypothyroid and 10 (9.3%) latent hyperthyroid. Secondary hyperparathyroidism was present in 19 (17.3%), 25-hydroxyvitamin D deficiency in 64 (54.7%) of patients. Osteoporosis was present in 26 (21.1%), osteopenia in 59 (48.0%) patients. 47 vertebral fractures, 3 hip and 1 humerus fractures occurred in 21 patients. Most of the patients had coincidence of two or three disorders, while less than 5% did not have any endocrine irregularities. All patients received calcium and vitamin D supplements. Forty-six patients (37.4%) were treated with zoledronic acid, 12 (9.8%) with oral bisphosphonates. Two patients were treated with teriparatide.

**Conclusions:**

The prevalence of multiple endocrine disorders early after heart transplantation is high. Assessment and management of increased fracture risk and all other potentially affected endocrine axes should be considered as a standard of care in this early period.

## Background

Improvement of survival after heart transplantation (HT) brings new challenges in recognizing and managing endocrine complications in the patient population [1]. The most dynamic period with the greatest changes in endocrine profile is the first post-transplant year [2–4] resulting in vitamin D deficiency, hypogonadism, post-transplant diabetes mellitus (PTDM), and bone loss [2–9].

Many factors have been associated with endocrine changes early after HT: association of glucocorticoid treatment with bone loss and vertebral fractures (VFs) is well established [10]. Treatment with a high-dose glucocorticoid regimen leads to accelerated bone loss in the first 6 months after HT, after which the rate of decline of bone loss slows down. Bone loss is predominantly trabecular [10]. In animal models, the calcineurin inhibitors (CNIs) cyclosporine A and tacrolimus are further associated with bone loss and increased bone turnover [11]. There is also evidence of CNI-associated bone loss in clinical settings, yet it is difficult to assess their separate impact on post-transplant osteoporosis since, in the early period, CNIs are always administered with glucocorticoids [12].

Furthermore, glucocorticoids and CNIs both cause hyperglycemia and PTDM [13]. High doses of glucocorticoids are also associated with hypogonadism, with the lowest levels of testosterone being observed immediately after HT [3]. Secondary hyperparathyroidism as a consequence of reduced kidney function, and decreased 25-hydroxycholecalciferol (25OHD) is also expected in the early post-transplant period. Low 25OHD is related to decreased dietary intake of 25OHD-containing foods, limited sun exposure and the effect of glucocorticoids on absorption and metabolism of 25OHD [14, 15]. Reduced kidney function is related to the underlying cardiac disorder (cardio-renal syndrome) and treatment with CNI [16]. The prevalence of secondary hyperparathyroidism after HT, ranging from 30 to 75%, has been assessed in only a few studies [6, 17, 18]. Similarly, thyroid function in the early post-transplant period has remained largely unaddressed [19–21].

## Methods

### Aims

The aim of this study was to assess the endocrine profile and endocrine management in a well-defined national cohort of heart transplant recipients in the early post-transplant period. We determined glucose metabolism, male hypogonadism, thyroid function, calciotropic axis, BMD, incidence of osteoporotic fracture and related treatment intervention.

### Study design, setting of the study, patient population

We conducted a retrospective cohort study on 123 consecutive HT recipients who received transplants at the Advanced Heart Failure and Transplantation Programme, Department of Cardiology, University Medical Center Ljubljana between the years 2009 and 2018. After discharge, all HT recipients were referred to the Endocrine Outpatient Clinics, Department of Endocrinology, Diabetes and Metabolic Diseases, University Medical Center Ljubljana for protocol-based endocrinological screening. Patients included in this analysis had at least one endocrine follow-up visit within the 1st post HT year. We excluded patients who failed to complete the endocrine outpatient follow-up visit within the 1st post-transplant year, pediatric patients (< 18 years of age) and patients with major postoperative non-fatal complications including post-operative renal replacement therapy or tracheostomy. Altogether, 39 patients were excluded: 37 of those patients had major postoperative complications (21 non-fatal, 16 fatal); 2 patients were pediatric (< 18 years of age).

At the time of endocrine assessment, all patients were treated with standard triple immunosuppression therapy (steroids, CNI, mycophenolate mofetil). The maintenance dose of methylprednisolone in all patients was 4 mg per day. The doses of cyclosporine and tacrolimus were regularly monitored and adjusted to target serum concentrations (cyclosporine A 200–250 μg/L; tacrolimus was 6–10 μg/L). The maintenance dose of mycophenolate mofetil in patients treated with cyclosporine A was 3000 mg per day and in patients treated with tacrolimus, 2000 mg per day. Per protocol, all HT recipients additionally received vitamin D (cholecalciferol and alphacalcidiol) and Ca^2+^ supplementation (Ca^2+^ carbonate 1 g per day).

In all patients, we collected epidemiological and transplant-related data and data on patients’ medical therapy. Specifically, we focused on collecting data on vitamin D deficiency, bone mineral density (BMD), history of low energy fractures, hypogonadism (in male recipients), PTDM, thyroid and parathyroid function. Normal endocrinological profile was defined as normal testosterone in men, normal TSH, iPTH, serum calcium, glucose homeostasis, sufficient concentration of 25OHD, normal BMD, and no fractures.

### Assessment of anthropometric parameters

Height was measured with an accuracy of 1 cm and body weight with an accuracy of 1 kg. Body mass index (BMI) was calculated as the weight in kilograms divided by square of height in meters.

### Biochemical analysis

We collected data for testosterone, free testosterone, sex hormone binding globulin (SHBG), thyroid-stimulating hormone (TSH), thyroxine (fT4) and triiodothyronine (fT3), intact parathyroid hormone (iPTH), corrected calcium, 25OHD, collagen type 1 cross-linked C-telopeptide (CTX), N-terminal propeptide of procollagen type 1 (P1NP), glucose, creatinine and estimated glomerular filtration rate (eGFR) calculated with Modification of Diet in Renal Disease Study equation [22]. Renal dysfunction was defined according to KDIGO guidelines [23].

### Assessment of calcium metabolism and bone-related disorders

Primary hyperparathyroidism is defined as corrected calcium increased above the upper level of normal (2.65 mmol/L) or in the upper half of normal (above 2.45 mmol/L) and iPTH increased above 65 pmol/L. Secondary hyperparathyroidism is reported if calcium decreases below the lower level of normal value or in the lower half of normal (lower than 2.45 mmol/L) and iPTH increases above 65 pmol/L [24].

Normal levels of 25OHD are defined as serum levels above 75.0 nmol/L. Insufficiency of 25OHD is defined as serum level 50.0–74.9 nmol/L, mild deficiency as 25.0–49.9 nmol/l and severe deficiency below 24.9 nmol/L. BMD was measured in the lumbar spine, hip and femoral neck by dual-energy X-ray absorptiometry (DXA) with the use of Discovery DXA System (Hologic, Bedford, MA). Measurements are provided in g/cm^2^ and T-scores. Osteoporosis is defined as T-score ≤ − 2.5 SD, osteopenia as − 1.0 SD < T-score > − 2.5 SD and normal BMD as T-score ≥ − 1.0 SD at any measured site. Osteopenia and osteoporosis are defined in accordance with the International Osteoporosis Foundation [25]. Osteoporotic VFs were confirmed by X-ray in patients with suspected fractures based on history and medical examination. Non-VFs were recorded based on history and medical records. Numbers of patients who were treated with intravenous or oral bisphosphonates (BP) or teriparatide within the 1st year post HT were recorded.

### Hypogonadism assessment

Testosterone was measured using coated tube RIA (DiaSorin S. p. A., Salluggia, Italy and Diagnostic Products Corporation, LA) and SHBG with chemiluminescent immunoassay method (Immulite 2000 Analyzer, Siemens Healthcare, Erlangen, Germany). Low levels of total testosterone were defined as testosterone serum levels of less than 11.0 nmol/L [26]. Hypogonadism was defined as testosterone deficiency with clinical signs of hypogonadism.

### Assessment of thyroid function

TSH was measured with the immune method of anti-FITC monoclonal antibody (ADVIA Centaur XP, Siemens Healthcare, Erlangen, Germany), fT3 and fT4 with the chemiluminescent immunoassay method (ADVIA Centaur XP, Siemens Healthcare, Erlangen, Germany), iPTH with the chemiluminescent immunoassay method (IMMULITE® 2000, Siemens Healthcare, Erlangen, Germany) and calcium with the CPC method (ADVIA 2400, Siemens Healthcare, Erlangen, Germany). Free T4 and fT3 were measured only in cases of high or low TSH. Normal levels of TSH were defined as 0.55–4.78 mE/L, fT4 11.5–22.7 pmol/L, fT3 4.6 pmol/L. Hypothyroidism was interpreted as increased TSH with normal or reduced fT3 and fT4 and as substitution of levothyroxine [27]. Hyperthyroidism was interpreted as treatment with thyreostatic drugs or reduced TSH with normal or increased fT3 and fT4 [28].

### Glucose metabolism assessment

Glucose was measured with the standard oxidase method (Beckman Coulter Glucose Analyzer, Beckman Coulter Inc. CA, USA). The diagnosis of post-transplant diabetes mellitus (PTDM) was defined as newly diagnosed fasting glucose ≥7.0 mmol/L on more than one occasion, random glucose ≥11.1 mmol/L with symptoms, two-hour glucose after a 75 g oral glucose tolerance test of ≥11.1 mmol/L, and HbA1c ≥6.5% [29]. We identified patients with diabetes mellitus (DM) and defined type of DM in each individual. We acquired information about anti-diabetic medical therapy and/or any changes in the anti-diabetic medical regimen after HT from institutional patient digital records.

### Statistical methods

All statistical analyses were done for descriptive purposes. Numerical variables are presented as median (interquartile range), and categorical variables with proportions. 95% binominal (Clopper-Pearson) exact confidence intervals for proportions were computed. We used the Mann-Whitney test to compare differences in gender, age and treatment with zoledronic acid (ZA). Correlation of iPTH with eGFR, level of chronic kidney disease and 25OHD was calculated with Spearman’s rank correlation coefficient. *P* value of < 0.05 was considered statistically significant. Statistical analyses were performed using IBM SPSS Statistics for Windows, Version 24.0 (IBM Corp., Armonk, NY).

## Results

Of 123 patients included in the analysis, 22 (17.9%) were women and 101 (82.1%) were male. The median time to first post HT visit to the endocrinology outpatient clinic was 3 months (IQR 2–4). Demographic, anthropometric, hormonal biochemical and densitometric baseline characteristics of the analyzed cohort are presented in Table 1.

**Table 1:** Demographic, anthropometric hormonal, biochemical and densitometric characteristics of the cohort

	Median	IQR
**Age** (years)	57	50–63
**Weight** (kg)	74.0	64.5–82
**Height** (cm)	174.0	168.0–180.0
**BMI** (kg/m^2^)	24.25	22.75–26.00
**cCa** (mmol/L)	2.27	2.20–2.33
**25OHD** (nmol/L)	73.5	54.65–89.55
**iPTH** (pmol/L)	35.3	24.525–50.775
**Creatinine** (μmol/L)	79	69–97
**eGFR** (mL/min/1.73 m^2^)	81	68–90
**Testosterone** (nmol/L)	14.9	12.40–21.95
**TSH** (mE/L)	1.45	1.09–2.10
**CTX** (pmol/L)	4377	3292–6415
**PINP** (μg/L)	30.2	20.9–49.0
**BMD lumbar spine** (g/cm^2^)	0.951	0.866–1.043
**T-score lumbar spine** (SD)	−1.1	−2.0- - 0.3
**BMD femoral neck** (g/cm^2^)	0.778	0.667–0.879
**T-score femoral neck** (SD)	−1.1	− 1.9- -0.3
**BMD hip** (g/cm^2^)	0.962	0.855–1.064
**T-score hip** (SD)	−0.5	−1.1-0.2

Legend: Data are median and interquartile range (IQR). *Time form TX* time from transplantation, *cCa* corrected calcium, *CTX* C-terminal telopeptide, *25OHD* 25 hydroxyvitamin D, *iPTH* intact parathyroid hormone, *eGFR* estimated glomerular filtration, *PINP* procollagen type I N-terminal propeptide, *TSH* thyroid-stimulating hormone, *SD* standard deviation

### Diabetes mellitus

Prevalence of diabetes mellitus is presented in Table 2. 39 (31.7%) of the included patients had DM or PTDM. In patients who were diagnosed with DM prior to HT, the antidiabetic therapy was intensified in the early period after HT for 18 (14.6%), and 3 (2.4%) patients had the same treatment in the period between HT and first follow-up at the diabetes clinics. There was no data about antidiabetic intensification for 4 (3.3%) patients. Among the patients with type 2 DM, 1 (0.8%) was treated with a combination of sulfonyl urea and metformin, 2 (1.6%) were treated with biphasic, 2 (1.6%) with basal, 5 (4.1%) with prandial, 1 (0.8%) with combination of biphasic and prandial and 14 (11.4%) with a combination of basal and prandial insulin. 14 (11.4%) patients that did not have DM prior to HT were diagnosed with PTDM after HT. Among these patients 2 (1.6%) were prescribed diet only, 3 (2.4%) were treated with repaglinide, 1 (0.8%) with combination of repaglinide and metformin, 2 (1.6%) with biphasic, 3 (2.4%) with prandial insulin, 3 with combination of basal and prandial insulin.

**Table 2:** Prevalence of diabetes mellitus, hypogonadism, thyroid dysfunction, secondary hyperparathyroidism, disturbed calcium level, low bone mineral density and 25hydroxyvitamin D in early post-heart transplant period

Endocrine abnormality	Number of patients	Percentage of sample (95% CI)
**Diabetes Mellitus**		
Type 1	0	0 (−)
Type 2	25	20.3 (13.6–28.5)
PTDM	14	11.4 (6.4–18.4)
**Hypogonadism**	38	40.4 (30.4–51.0)
**Thyroid disfunction**		
Hypothyroidism	5	4.6 (1.5–10.5)
Hyperthyroidism	10	9.3 (4.6–16.4)
**Secondary hyperparathyroidism**	19	17.3 (10.7–25.7)
**Calcium status**		
Hypocalcaemia	5	4.1 (1.3–9.3)
Hypercalcaemia	2	1.6 (0.2–5.8)
**Low BMD**		
Osteoporosis	26	21.1 (14.3–29.4)
Osteopenia	59	48.0 (38.9–57.2)
**25OHD status**		
Normal	53	45.3 (36.1–54.8)
Mild deficient	39	33.4 (24.9–42.6)
Deficient	19	16.2 (10.1–24.2)
Insufficient	6	5.1 (1.9–10.8)

Legend: *PTDM* post transplant diabetes mellitus, *BMD* bone mineral density; *25OHD* 25hydroxyvitamin D, *CI* confidence interval

### Hypogonadism

In the first posttransplant year, testosterone was assessed in 94 male HT recipients (Table 2). Testosterone substitution with 10% testosterone gel was started in 17 (18.1%) patients with diagnosed hypogonadism. Patients who received testosterone substitution reported substantially improved quality of life 3–6 months after the intervention. In 21 (22.3%) male recipients with hypogonadism, testosterone substitution was not started: in 14 (14.9%) because of mild clinical manifestation of hypogonadism and expected spontaneous improvement, in 4 (3.3%) due to increased prostate specific antigen, and in 3 (2.4%) as a result of the patient’s personal preference.

### Thyroid function

Data on TSH serum levels and levothyroxine substitution thyroid function was evaluated in 108 (87.8%) patients (Table 2). 10 (9.3%) hyperthyroid patients had latent hyperthyroidism and didn’t need specific treatment. 4 (3.7%) hypothyroid patients received substitution with levothyroxine. All hypothyroid patients received well controlled substitution of levothyroxine. In 35 (28.5%) patients, fT3 was measured and only 3 (8.6%) had values below normal.

### Calcium metabolism

Calcium metabolism, vitamin D status and prevalence of secondary hyperparathyroidism are presented in Table 2. All 123 (100%) patients received calcium substitution consisting of 1 g of calcium carbonate per day (in 4 (3.3%), treatment was initiated before transplantation and in 119 (96.7%), during hospitalization after transplantation) and in median, 0.5 μg alfacalcidol per day (3 (2.4%) before transplantation, 114 (92.7%) in hospitalisation after transplantation and 6 (4.9%) in the first year after transplantation) and 116 (94.3%) cholecalciferol in median 14,000 IU per week in addition to alfacalcidiol (32 (27.6%) before transplantation, 71 (61.2%) in hospitalization after transplantation and 13 (11.2%) in the first year of transplantation). There was a weak negative correlation between iPTH and eGFR (− 0.224; *p* < 0.05), weak positive correlation between level of chronic kidney disease (0.210; *p* < 0.05) and no correlation between iPTH and 25OHD serum levels.

### Bone-related disorders

BMD on the lumbar spine, hip, and/or femoral neck were measured in all 123 patients (Table 2). There was a significant difference in lumbar spine BMD (*p* = 0.026), femoral neck BMD (*p* < 0.001), femoral neck T-score (*p* = 0.006), hip BMD (*p* < 0.001), hip T-score (*p* = 0.003) between male and female patients, with male patients presenting with higher BMD and T score at all measured sites. Stratifying patients according to their age, no difference in BMD were observed at any of the measured sites, regarding BMD and T-scores for lumbar spine, hip, femoral neck and distal radius groups ≤49 years (27 patients (22.0%)), 50–59 years (47 patients (38.2%)), ≥60 years (49 patients (39.8%)).

In terms of fractures, 21 (17.1%) patients altogether sustained 49 VF: 3 hips, 2 humerus and 1 wrist fracture, among which 2 VF, 1 humerus and wrist fracture were already documented before HT. Within the first posttransplant year, 47 VFs, 3 hip fractures and 1 humerus fracture occurred in 16 patients. Ten VFs in 5 patients were clinically silent and 37 VFs in the remaining 11 patients were symptomatic. 24 VF, 2 hip, 1 humerus and 1 wrist fractures occurred in 9 patients with established osteoporosis, 19 VF, 1 hip and 1 humerus fracture occurred in 10 patients with osteopenia, and 6 VFs were established in patients with normal BMD. The most commonly fractured vertebrae after transplantation was Th11 (in 7 patients), followed by 6 fractures of Th12 and L1; 5 of Th8 and L2; 4 of Th7, Th9 and L3; 2 of Th10 and Th6 and 1 fracture of L4 and L5.

We based the decision for the initiation of osteoporosis treatment on the assessment of increased fracture risk including DXA, history of previous fractures, kidney function and comorbidities. Specific antiosteoporotic treatment in addition to calcium and 25OHD supplementation was indicated at endocrine outpatient follow-up in 85 (69.1%) patients. Median time from HT to first treatment with bisphosphonates was 7 months (IQR 4–9). 46 (37.4%) patients were treated with zoledronic acid and 12 (9.8%) patients with oral BP. Two patients (1.6%) were treated with teriparatide. When comparing patients who received zoledronic acid with the remaining cohort, we detected a significant difference in baseline BMD and T-scores for femoral neck, lumbar spine and eGFR (Table 3).

**Table 3:** Comparison between patients treated with ZA and patients not treated with ZA

Parameter	Treated with ZA (***N*** = 46)	Not treated with ZA (***N*** = 63)	Significance
**Lumbar spine (BMD)**	0.907 (0.866–0.978)	1.012 (0.933–1.118)	*p* < 0.001
**Lumbar spine (T-score)**	−1.6 (−2.2- -1.0)	−0.6 (−1.2–0.4)	*p* < 0.001
**Femoral neck (BMD)**	0.749 (0.680–0.813)	0.832 (0.757–0.946)	*p* = 0.002
**Femoral neck (T-score)**	−1.3 (− 1.8- -0.8)	−0.7 (− 1.3–0.1)	*p* = 0.002
**Hip (BMD)**	0.935 (0.860–1.007)	1.009 (0.925–1.094)	*p* = 0.006
**Hip (T-score)**	−0.7 (−1.1- -0.2)	0.0 (− 0.6–0.4)	*p* = 0.003
**eGFR**	90 (75–90)	77 (66–90)	*P* = 0.019

Legend: ZA was administered in median time 7 months from HT. Patients not treated with ZA are treatment naïve; patients who received peroral bisphosphonates and teriparatide were excluded from analysis. Data are median and interquartile range (IQR). *BMD* bone mineral density, *eGFR* estimated glomerular filtration, *ZA* zoledronic acid

### The coincidence of endocrine disorders

**Fig. 1:**
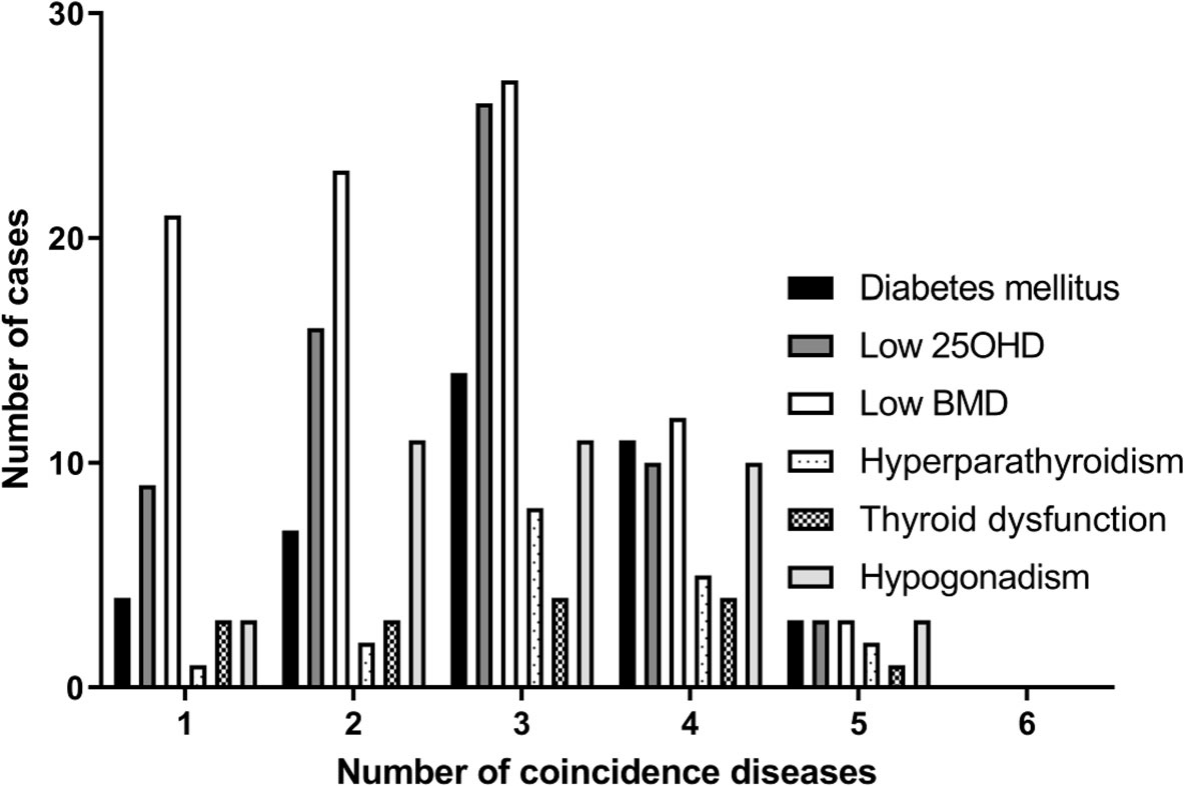
Coincidence of endocrine disorders. 25OHD – 25 hydroxyvitamin D; BMD – bone mineral density

The coexistence of endocrine disorders in our patient cohort is presented in Fig. 1. 41 (33.3%) patients had 1, 31 (25.2%) 2; 30 (24.4%) 3; 13 (10.6%) 4 and 3 (2.4%) had 5 established endocrine disorders. 5 (4.1%) patients did not have any endocrine disorders. None of the patients had all 6 disorders (Fig. 1).

### Kidney function

Kidney function was normal in 53 patients. There were 70 patients with CKD (52 with CKD stage 2 and 18 with CKD stage 3).

### Survival data

30 days, 3 and 12-month survival of our cohort was 93, 92 and 90% respectively. Due to low mortality rates, we did not analyze potential association between endocrine abnormalities and post-transplant survival.

## Discussion

The current study presents a thorough assessment of the endocrine profile of HT recipients in the early post-transplant period. Our main findings suggest that the 25OHD deficiency is most prevalent, followed by low BMD and hypogonadism in males. Other disorders such as diabetes mellitus, hyperparathyroidism and thyroid dysfunction are also common. Most patients had a coincidence of two or three disorders; less than 5% had no endocrine irregularities.

In our cohort, 11.4% of the patients developed PTDM within the first 12 months post HT. These results are in accordance with those provided by Ye et al. [5], where cumulative rates of PTDM were 11.1, 32.0 and 38.4% after 12-, 24- and 36-months post HT respectively [5]. With the documented time-dependent increase in incidence of PTDM after HT, it is clear that other factors in addition to glucocorticoids, in particular CNIs, are involved in its pathogenesis, and that this entity differs from steroid DM [30]. CNIs decrease insulin release by direct toxic effect on pancreatic β-cells. Cyclosporine A binds to cyclophilin D in the mitochondrial permeability transition pore and interferes with insulin stimulation. CNIs regulate the dephosphorylation of the nuclear factor of activated T-cell proteins and cAMP responsive element-binding transcription factor activity-2. This decreases β-cell survival, replication and function [13].

Many studies have reported that solid organ transplant recipients have low 25OHD serum levels [2, 31]. In our cohort, 25OHD deficiency was common (64 (54,7%)), despite substitution of cholecalciferol that had been started before or immediately after HT in most of the patients. In a study by Stein et al., 91% of transplant recipients had low 25OHD shortly after HT, which was more than two times higher than in our cohort. While Stein et al. used calcium supplements containing also 400–600 IU of cholecalciferol and multivitamin containing 400 IU cholecalciferol [2], our patients received in median 2000 IU of cholecalciferol per day and in median 0.5 μg alfacalcidol per day. Based on these data, we propose that treatment with cholecalciferol and/or alfacalcidiol should be started before transplantation and 25OHD serum levels should be carefully monitored throughout the duration of substitution. This is particularly relevant since vitamin D deficiency is related to significant post HT bone loss and fractures, muscle weakness and increased risk of falls [6] and might potentiate the immunosuppressive action of calcineurin inhibitors or prednisolone [32].

In our cohort, about 20% of patients had secondary hyperparathyroidism. The incidence of hyperparathyroidism in other studies on solid organ recipients was even higher, ranging between 30 and 100% [5, 17, 18]. The lower incidence of secondary hyperparathyroidism in our cohort is probably related to more intensive treatment with cholecalciferol, along with alfacalcidiol and calcium supplementation that had been started before or immediately after HT, and resulted in lower incidence of hypovitaminosis D than reported in other studies [5, 17, 18]. Interestingly, the pathophysiological mechanisms underlining the hyperparathyroidism in this patient cohort are not completely clear, as it was not related to liver or renal failure, low 25OHD or low 1.25-dihydroxycholecalciferol [17]. We speculate that mild impairment of kidney function is sufficient for an increase in iPTH in HT recipients when compared with the general population [5]. This hypothesis is in accordance with our data, as in our cohort, hyperparathyroidism was weakly negatively correlated to eGFR and weakly positively correlated with the level of chronic kidney disease.

In terms of thyroid disorders, recent studies suggest that hyperthyroidism is present in 21%, hypothyroidism in 13%, and low fT3 syndrome in 18 patients within 1 month after HT respectively [20]. Our results corroborate these data, as hyperthyrosis was established in 10% and hypothyrosis in 5% of the studied cohort. Clinical effects of thyroid disorders after HT are substantial, as hypothyroidism is linked to increased length of hospitalization, and cytomegaloviral infections occur most commonly in patients with hyperthyroidism [20]. Atrial fibrillation could be associated with hyperthyroidism, but was not observed in our patient cohort within the first post-HT year. The most important risk factor of thyroid disfunction in the HT population is a history of treatment with amiodarone [21]. History of treatment with amiodarone was not consistently available in our cohort, and was therefore not included in the analysis.

Hypogonadism is common in male post-transplant patients [33]. In our cohort, the incidence of hypogonadism was 40%, which is in accordance with other published studies. In a study by Fleischer, total testosterone was decreased in 63% of men after 1 month, in 33% 2 months and in 21% 6 months after HT [3]. It is suggested that hypogonadism persists in 14% at 1 year after transplantation [3]. It is currently assumed that treatment with corticosteroids has the most important impact on gonadal axis, followed by an influence of recent major surgery [34, 35]. Pathophysiological mechanisms of hypogonadism after HT remain underexplored. While the effect on direct inhibition of testicular testosterone synthesis and suppression of luteinizing hormone secretion is well established in animal studies, currently there is no data to suggest any negative effects of cyclosporine A on hypogonadism in the clinical setting. Low testosterone might also be a marker of impaired graft function and an increased incidence of low-grade rejection episode early after HT [36–38].

In HT patients, the most rapid bone loss associated with fractures develops during the 1st year after transplantation [4]. Mechanisms of bone loss in this period are multifactorial and include combinations of accelerated turnover and hypogonadism [7], low 25OHD, secondary hyperparathyroidism, dietary calcium deficiency and medication such as corticosteroids, loop diuretics, and CNIs [39]. Our results were in accordance with the study of Carbonare et al., where 180 HT patients had BMD measured on lumbar spine and hip. BMD was reduced in osteoporotic range in 13% on the spine and 25% on the hip [40].

17.1% of patients in our cohort were diagnosed with fractures, and 87% of VF and all non-VFs occurred in relation to osteoporosis and osteopenia. A study by Leidig-Bruckner on 105 HT patients reported the occurrence of at least one VF in 21% of patients within the first post HT year, which is higher than shown in our data [41]. Most of the other evidence, based on older data, shows 40–48.2% incidence of VFs in HT recipients [40, 42, 43], with T-score below − 1.5 SD being recognized as the most important risk factor [40]. The median T-scores in our patient cohort were − 1.1 SD for the lumbar spine, − 1.1 SD for the femoral neck and 0.5 SD for the hip. Most of our patients were treated with calcium substitution and vitamin D supplementation before or immediately after HT; 65% of patients received BP within 1st year, 54% of patients were treated with ZA, and 2 patients received teriparatide. Compared to a study published by Löfdahl, where 35, 38 and 16% received calcium supplements, vitamin D supplements and BP in the first postoperative year respectively [44], the number of treated patients in our cohort was significantly higher. ZA was recognized as the most effective antiresorptive drug for prevention of transplantation bone loss in this population [45]. Relatively higher BMD and intensive treatment intervention with the most efficient antiresorptive drug available for this population can explain the lower incidence of fractures in our patients when compared to previously reported observations that included patients in a comparable timeframe [40, 42]. However, even greater reduction of fractures could likely be achieved with preoperative treatment when decreased BMD is present. In a more recent cohort of 105 HT patients, where antiresorptive treatment was initiated before solid organ transplant or immediately post-transplant, the incidence of VFs in the 1st year was 3.8% [46]. It is thus important to follow the guidelines of The International Society of Heart and Lung Transplantation guidelines for the care of HT recipients which recommend BP treatment for all HT recipients with decreased BMD within the first post-transplant year [47].

Several limitations of our study need to be addressed. The major limitation of this study is its retrospective, single-center design, with missing data and limited availability of X-rays of the spine in asymptomatic patients, which can result in underreported clinically silent VFs. Nevertheless, our results were derived from a relatively large and a well-defined cohort of HT recipients, who were treated according to a strict and uniform immunosuppressive protocol which can significantly decrease selection bias. Additionally, this is the first study to analyze all endocrine axes in HT recipients in the early post-transplant period. With this we are confident that our results can add to the current knowledge of endocrine disorders in this patient cohort. Additionally, our results may aid in the design of future research for the endocrine management of HT recipients.

## Conclusion

In summary, endocrine disorders are highly prevalent in an early post HT period, particularly 25OHD deficiency, osteoporosis and hypogonadism in males. In order to reduce morbidity and mortality [48] and improve patients’ quality of life [49], assessment of endocrine profile in patients after HT, including glucose metabolism, hypogonadism, thyroid function, hyperparathyroidism, level of 25OHD, level of calcium, BMD, osteoporotic fracture, and selection of timely and appropriate interventions, is of paramount importance. PTDM needs long term follow up, since the incidence increases with the time from transplantation. The incidence of male hypogonadism within first post HT year is high. However, the treatment of hypogonadism should be individually tailored and probably reserved for individuals with severe clinical manifestation in particular, since a high rate of spontaneous remission of gonadal axis can be expected 6 months post transplantation. The etiology of secondary hyperparathyroidism in the early post HT period needs further exploration, since it might go beyond vitamin D deficiency and chronic kidney disease. Most HT patients developed thyroid illness in the first post HT year, a history of amiodarone administration before HT being the strongest risk factor. Although bone loss is a major determinant for fractures, there are other determinants that should be identified and treated. High incidence of clinically silent VFs encourages active periodic search for potential VFs by X-ray in all HT patients. The role of additional tools, such as trabecular bone score (TBS) assessment, that would better characterize the increased fracture risk in this population needs to be addressed in future research. The role of teriparatide should be defined in those with high risk of fractures or prevalent fractures, in particular multiple VFs. Early initiation of intensive fracture risk reduction, probably even before HT, is highly recommended.

## Data Availability

The data that support the findings of this study are available from the corresponding author upon reasonable request.

## Abbreviations

25OHD: 25-hydroxycholecalciferol
BMD: Bone mineral density
BMI: Body mass index
BP: Bisphosphonates
CNIs: Calcineurin inhibitors
CTX: Collagen type 1 cross-linked C-telopeptide
DXA: Dual-energy X-ray absorptiometry
eGFR: estimated glomerular filtration rate
fT3: triiodothyronine
fT4: thyroxine
HT: Heart transplantation
iPTH: intact parathyroid hormone
P1NP: N-terminal propeptide of procollagen type 1
PTDM: Post-transplant diabetes mellitus
SHBG: Sex hormone binding globulin
TSH: Thyroid-stimulating hormone
VFs: Vertebral fractures
ZA: Zoledronic acid
